# Combination therapy of human bone marrow–derived mesenchymal stem cells and minocycline improves neuronal function in a rat middle cerebral artery occlusion model

**DOI:** 10.1186/s13287-018-1011-1

**Published:** 2018-11-09

**Authors:** Dong Young Cho, Sin-Soo Jeun

**Affiliations:** 1 Department of Neurosurgery, Seoul St. Mary’s Hospital, The Catholic University of Korea, 222 Banpo-daero, Seocho-gu, Seoul, 137-701 Korea; 2 Department of Biomedical Science, College of Medicine, The Catholic University of Korea, 222 Banpo-daero, Seocho-gu, Seoul, 137-701 Korea

**Keywords:** Cerebral ischemia, Human bone marrow–derived mesenchymal stem cell, Minocycline

## Abstract

**Background:**

The positive effects of human bone marrow–derived mesenchymal stem cells (hBM-MSCs) and minocycline on ischemic stroke models have been well described through numerous studies. The aim of this study was to evaluate the effectiveness of combination therapy of hBM-MSCs with minocycline in a middle cerebral artery occlusion rat model.

**Methods:**

Forty male Sprague-Dawley rats were enrolled in this study. After right middle cerebral artery occlusion, rats were randomly assigned to one of four groups: control, minocycline, hBM-MSCs, or hBM-MSCs with minocycline. Rotarod test, adhesive-removal test, and modified neurological severity score grading were performed before and 1, 7, 14, 21, and 28 days after right middle cerebral artery occlusion. All rats were sacrificed at day 28. The volume of the infarcted area was measured with triphenyl tetrazolium chloride staining. Neuronal nuclear antigen (NeuN)- and vascular endothelial growth factor (VEGF)-positive cells in the ischemic boundary zone were assessed by immunofluorescence.

**Results:**

Neurological outcome in the adhesive-removal test and rotarod test and modified neurological severity score were better in the combination therapy group than in the monotherapy and control groups. The volume of the infarcted area was smaller in the combination group compared with the others. The proportions of NeuN- and VEGF-positive cells in the ischemic boundary were highest in the combination therapy group.

**Conclusions:**

Early combination therapy of hBM-MSCs with minocycline in an ischemic stroke model may enhance neurological recovery, reduce the volume of the infarcted area, and promote the expression of NeuN and VEGF in ischemic boundary cells.

**Electronic supplementary material:**

The online version of this article (10.1186/s13287-018-1011-1) contains supplementary material, which is available to authorized users.

## Background

The population of patients who have ischemic disease of the brain has risen along with greater life expectancy and changes in lifestyle. Recent treatment strategies for ischemic stroke are focused on prevention, such as lifestyle modification, use of an antiplatelet agent, intraluminal angioplasty, or bypass surgery. Treatment options after ischemic stroke are very limited because of the irreversibility of neuronal tissue damage. To overcome those difficulties, numerous studies are ongoing.

Stem cell therapy for ischemic stroke is emerging as a fundamental treatment. Among the various kinds of stem cells, human bone marrow–derived mesenchymal stem cells (hBM-MSCs) are most frequently used for ischemic stroke because of the ease of harvesting and incubation, low immunogenicity, and the high potency of neuromodulation [1]. Owing to the low immunogenic profile of hBM-MSCs, their xenogeneic transplantation is known to be safe and effective [2, 3].

When hBM-MSCs are transplanted, they show therapeutic effects through migration, neural circuit reconstruction, angiogenesis, neurotrophic factor secretion, apoptosis inhibition, and immunomodulation [4–8]. Microglia and astrocytes in the infarcted area secrete stromal cell–derived factor 1, which interacts with chemokine receptor 4 expressed on hBM-MSCs, inducing migration [9]. Migrated hBM-MSCs enhance axonal plasticity, repair neural networks, and reconstruct neural circuits. These restoration processes contribute to the improvement of sensorimotor functions [10]. Transplanted hBM-MSCs also induce angiogenesis by secreting vascular endothelial growth factor (VEGF), placental growth factor, and basic fibroblast growth factor and maintaining the integrity of the blood–brain barrier (BBB) through crosslinking [11, 12]. Neurotrophic factors such as epidermal growth factor, brain-derived neurotrophic factor, basic fibroblast growth factor, nerve growth factor, insulin-like growth factor 1, glial cell line–derived neurotrophic factor, hepatocyte growth factor, and stem cell factor are secreted in hBM-MSCs, facilitating astrocyte proliferation and enhancing the efficacy of neuronal synapses [13]. hBM-MSC transplantation also reduces the Bax/Bcl-2 ratio and inhibits caspase-3 activity, thereby inhibiting apoptosis [14]. Immunomodulatory mechanisms of hBM-MSCS in the ischemic brain include inhibition of proliferation of T cells, macrophages, B cells, natural killer cells, and antigen-presenting cells, which result in suppression of inflammatory reactions to minimize neural cell damage [15]. Research into other mechanisms is ongoing. hBM-MSC transplantation at the acute phase of ischemic stroke protects neuronal cells through BBB stabilization and immunomodulation but is also effective even if hBM-MSCs are transplanted long after the acute phase, through angiogenesis and amplifying axonal plasticity [16].

Minocycline is a semisynthetic tetracycline analogue that has been used as a broad-spectrum antibiotic to treat Gram-positive and Gram-negative cocci infections for decades. It is also known to be effective in neurological disorders because of its anti-inflammatory and anti-apoptotic effects and to have excellent BBB penetration. Diverse studies have shown the therapeutic effects of minocycline in various central nervous system (CNS) disorders such as ischemic stroke, Parkinson’s disease, Huntington’s disease, and multiple sclerosis [17, 18]. Minocycline blocks nitric oxide–induced neurotoxicity by inhibiting P38 mitogen-activated protein kinase [19], decreases glutamate toxicity [20], and inhibits activation and proliferation of microglia [21], leading to anti-inflammatory effects. Minocycline also shows anti-apoptotic effects by reducing caspase-1 activation and inhibiting cytochrome C release [22]. Additionally, minocycline exerts a neuroprotective effect by inhibiting matrix metalloproteinase activity, which promotes apoptosis and disruption of the BBB in focal cerebral ischemia [23]. Based on these diverse neuroprotective effects, numerous studies have been performed with minocycline as a therapeutic agent for cerebral ischemia. Various *in vivo* studies with animals have shown reduced infarction size and improvement of neurologic deficits in ischemic stroke treated with minocycline [24, 25].

We previously reported the effectiveness of combination therapy with minocycline and hBM-MSCs in mice with autoimmune encephalomyelitis [17]. In this study, we examined the effect of combination therapy on cerebral ischemic disease in a middle cerebral artery occlusion (MCAO) rat model.

## Methods

### Preparing hBM-MSCs

hBM-MSCs (Catholic MASTER cells) were obtained from the Catholic Institute of Cell Therapy (CIC) (Seoul, Korea). Bone marrow aspiration was performed on the iliac crest of healthy donors who were 20 to 55 years old. These donations were approved by the institutional review board of Seoul St. Mary’s Hospital (Seoul, Korea). Of the donated bone marrows from different donors, one was selected and delivered to the Good Manufacturing Practice (GMP)-compliant facility of the CIC, and isolation, proliferation, and quality control of hBM-MSCs were performed. The marrow pellet was obtained by centrifuging aspirated bone marrow at 4 °C and 793*g* for 7 min. After centrifugation, a 10-fold volume of sterile distilled water was added and suspended to remove red blood cells (RBCs). RBC-deprived marrow pellets were suspended in MSC growth medium (GE Healthcare, Seoul, Korea) with 20% fetal bovine serum (GE Healthcare) and added to T-75 tissue culture flasks (Nunc, Rochester, NY, USA). The flasks were placed in an incubator for culture at 37 °C with 5% CO_2_. MSC growth medium was used for all cell expansion procedures and was replaced twice per week. Cells were detached when they reached about 70% to 90% confluence and re-plated at a density of about 5 to 8 × 10^3^ cells/cm^2^. Cells were expanded 2 to 4 passages in the GMP-compliant facility. Mycoplasma sterility, bacterial sterility, and endotoxin level (< 3 EU/mL) were tested during cell expansion. Additionally, cellular surface antigens (CD90/CD73, >95% positive; CD34/CD45, >95% negative) and multidifferentiation potential were tested for cells after the fourth passage.

### Preparing minocycline

Minocycline was purchased from Sigma-Aldrich (St. Louis, MO, USA) as a powder, dissolved in distilled water at 1 mM, and sterilized with a filter.

### Preparation of MCAO rat models

Adult male Sprague-Dawley rats (OrientBio, Seungnam, Korea) weighing about 270 to 400 g were used for the stroke model. Right MCAO was performed by intraluminal vascular occlusion with the monofilament method [26]. Rats were initially anesthetized by confinement to a sealed box with 5% isoflurane, and general anesthesia was maintained by facial mask with 1.5% isoflurane and a mixture of 70% N_2_O and 30% O_2_. Body temperature was measured by rectal measurement and maintained at 37 °C by using a heating pad (Panlab S.L., Barcelona, Spain). In the supine position, bilateral arms, legs, and tail were fixed on a plate. A linear midline vertical skin incision was made on the neck above the sternal notch after the hair was shaved. After dissecting the right sternocleidomastoid and omohyoid muscles, exposure of the right common carotid artery and carotid bifurcation were achieved. Ligation of the common carotid artery, external carotid artery, and pterygopalatine artery was carried out with 5–0 silk. After that, the bilateral tips of a 20-mm-sized 4–0 monofilament were heated near an alcohol lamp flame, producing oval-shaped tips. A tiny incision was made with the sharp needle tip on the common carotid artery, and the monofilament was introduced through the common carotid artery to the internal carotid artery and advanced 20 mm to locate the tip of the monofilament on the proximal middle cerebral artery (MCA). Internal carotid artery ligation followed monofilament introduction and skin suture. Transcranial Doppler (Esaote, Genova, Italy) was performed, and a more than 50% decrease in blood flow velocity of the right MCA compared with the left MCA was defined as proper MCAO after the procedure. All animal experiments were approved by the Institutional Animal Care and Use Committee of the Catholic University of Korea, College of Medicine.

### Transplantation of hBM-MSCs and administration of minocycline

For the study, 40 MCAO rat models were randomly assigned to one of four groups: control (n = 10), minocycline (n = 10), hBM-MSCs (n = 10), or minocycline with hBM-MSCs (n = 10). In the control group, an intraperitoneal injection of distilled water (1 mL) was carried out 24 h after MCAO and repeated every 24 h for 14 days. An intravenous phosphate-buffered saline (PBS) injection (100 μL) was carried out 72 h after MCAO via the tail vein. In the minocycline group, intraperitoneal injection of minocycline (5 g/mL solution, 20 mg/kg) was carried out 24 h after MCAO and repeated every 24 h for 14 days. An intravenous PBS injection (100 μL) was carried out 72 h after MCAO via the tail vein. In the hBM-MSC group, intraperitoneal injection of distilled water (1 mL) was carried out 24 h after MCAO and repeated every 24 h for 14 days. An intravenous injection of hBM-MSCs (1.5 × 10^6^ cells in 100 μL PBS) was carried out 72 h after MCAO via the tail vein. In the minocycline with hBM-MSC group, intraperitoneal injection of minocycline (5 g/mL solution, 20 mg/kg) was carried out 24 h after MCAO and repeated every 24 h for 14 days. Intravenous injection of hBM-MSCs (1.5 × 10^6^ cells in 100 μL PBS) was carried out 72 h after MCAO via the tail vein. All rats were sacrificed at day 28.

### Behavior tests and neurologic examination

All behavior tests and neurologic examinations were performed before MCAO and 1, 7, 14, 21, and 28 days after MCAO. All behavior tests were repeated 3 days before MCAO for adaptations.

#### Rotarod test

To estimate motor functions of the rats, a rotarod test with acceleration (Ugo Basile, Varese, Italy) was performed. The rats were positioned on a rotarod cylinder, and the spinning rate was accelerated from 4 to 40 rpm within 5 min. The test ended when the rats fell from the rung or hung on the rung while grasping the rod for two revolutions. The time from start to end of trial was measured. The trial was repeated three times, and the average value was determined. The percentage of recovery compared with pre-MCAO was calculated and recorded.

#### Adhesive-removal test

Adhesive-removal tests were performed to estimate sensorimotor functions [27]. Adhesive paper dots were stuck on both forepaws of the rats for tactile stimuli. The rats were located in the cage in a calm mood and observed until they removed the adhesive paper dots by using their teeth. The time from application of the paper dots until removal from the paws was recorded to a maximum of 3 min. All tests were repeated three times, and the mean value was obtained.

#### Modified neurological severity score

Modified neurological severity score (mNSS) grading was performed for neurological assessment [27]. Motor, sensory, balances, and reflexes were measured and graded from 0 to 18. A lower score indicated a favorable neurological status, and a higher score represented severe neurologic deficits.

### Measuring the volume of infarction

Twenty-eight days after MCAO, 5 out of 10 rats in each of the four groups were sacrificed and their brains were removed and sliced into 2-mm-thick coronal sections from bregma by using a rat brain mould. Sliced brain sections were soaked in a 2% solution of 2,3,5-triphenyl tetrazolium chloride (Sigma-Aldrich) with PBS for 30 min at a temperature of 37 °C. The volume of infarction in each coronal section was measured by MetaMorph image analyzing software (Molecular Devices, San Jose, CA, USA). To avoid overestimation of infarction volume due to brain edema, the corrected infarction volume (CIV) was obtained by the following formula:

$$ \mathrm{CIV}=\left[\mathrm{LT}-\left(\mathrm{RT}-\mathrm{RI}\right)\right]\times d, $$

where LT is the volume of the left hemisphere, RT is the volume of the right hemisphere, RI is the volume of the infarcted area, and *d* is the slice thickness (in millimeters) [28]. Total CIV was calculated by the sum of all slices, and the percentage of total infarcted volume was measured.

### Assessment of NeuN and VEGF expression

Twenty rats were sacrificed 28 days after MCAO for immunohistochemistry. After sacrifice with CO_2_ gas, the thoracic cage of the rats was opened and PBS with 4% paraformaldehyde was perfused intracardially for fixation. The brain was excised 24 h after postfix and immersed in a 30% sucrose solution for another 24 h. The fixed brain tissue was embedded with liquid nitrogen and frozen in − 80 °C. Frozen brain tissue blocks were coronally cryosectioned to a 10-μm thickness and embedded on slide glasses.

Neuronal nuclear antigen (NeuN) and VEGF expression was assessed by evaluating the ratio of NeuN- and VEGF-positive cells in the ischemic boundary zone. Anti-NeuN and anti-VEGF antibodies (Chemicon, Temecula, CA, USA) were applied as primary antibodies in double-tissue staining. Secondary staining was performed with Cy3-conjugated anti-mouse and anti-rabbit antibodies (Jackson ImmunoResearch Laboratories, Inc., West Grove, PA, USA) for visualization. Whole sections were counterstained with 4′,6-diamidino-2-phenylindole (DAPI) (Sigma-Aldrich). All images were captured with an LSM 700 confocal microscope (Carl Zeiss, Oberkochen, Germany). The proportion of NeuN- and VEGF-positive cells to DAPI counterstained cells in the ischemic boundary zone was calculated.

### Statistical analysis

All statistical analysis was carried out by SPSS Statistics version 20 (IBM, Armonk, NY, USA). One-way analysis of variance (ANOVA) with *post hoc* Bonferroni corrections and Kruskal–Wallis test were used for comparisons between the four groups. *P* values of less than 0.05 were considered to be statistically significant.

## Results

### Functional outcomes

#### Rotarod test

**Fig. 1:**
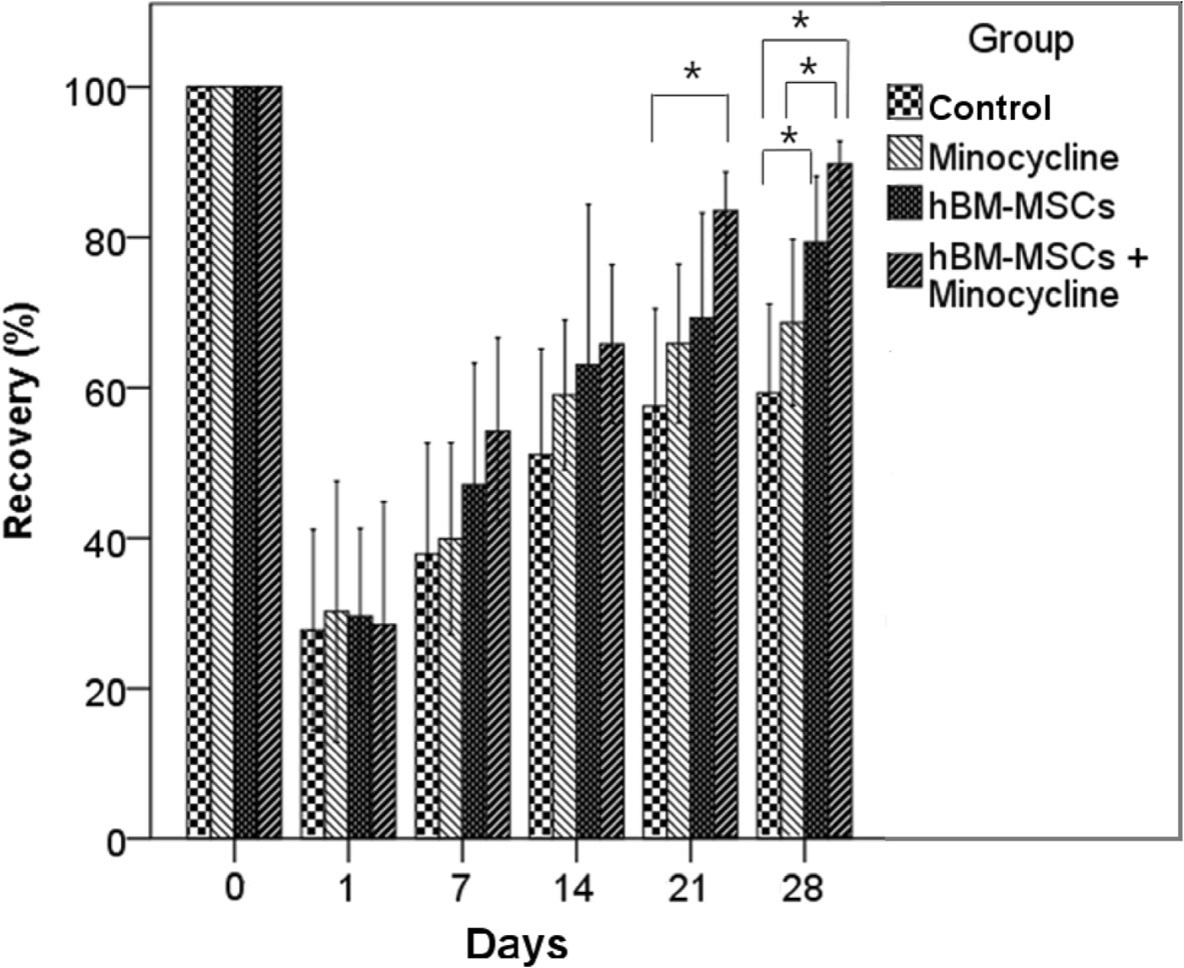
Rotarod test. Results of rotarod tests before middle cerebral artery occlusion (MCAO) and 1, 7, 14, 21, and 28 days after MCAO. The combination therapy group showed significant improvement compared with the control group 21 days after MCAO. At day 28, the combination group showed significant improvement compared with the control and minocycline groups. *P <0.05. Abbreviation: *hBM-MSC* human bone marrow–derived mesenchymal stem cell

The rotarod test did not show significant difference between the four groups until 14 days after MCAO. At 21 days after MCAO, however, the combination therapy group (83.5 ± 8.1%) showed a significant improvement in running time compared with the control group (57.6 ± 20.6%). At 28 days after MCAO, the hBM-MSC group (79.4 ± 13.9%) and combination therapy group (89.8 ± 4.8%) showed significant functional recovery compared with the control group (59.3 ± 18.7%). The combination therapy group also showed significant improvement of motor functions compared with the minocycline group (68.7 ± 17. 4%) (Fig. 1, Additional file 1: Table S1).

#### Adhesive-removal test

**Fig. 2:**
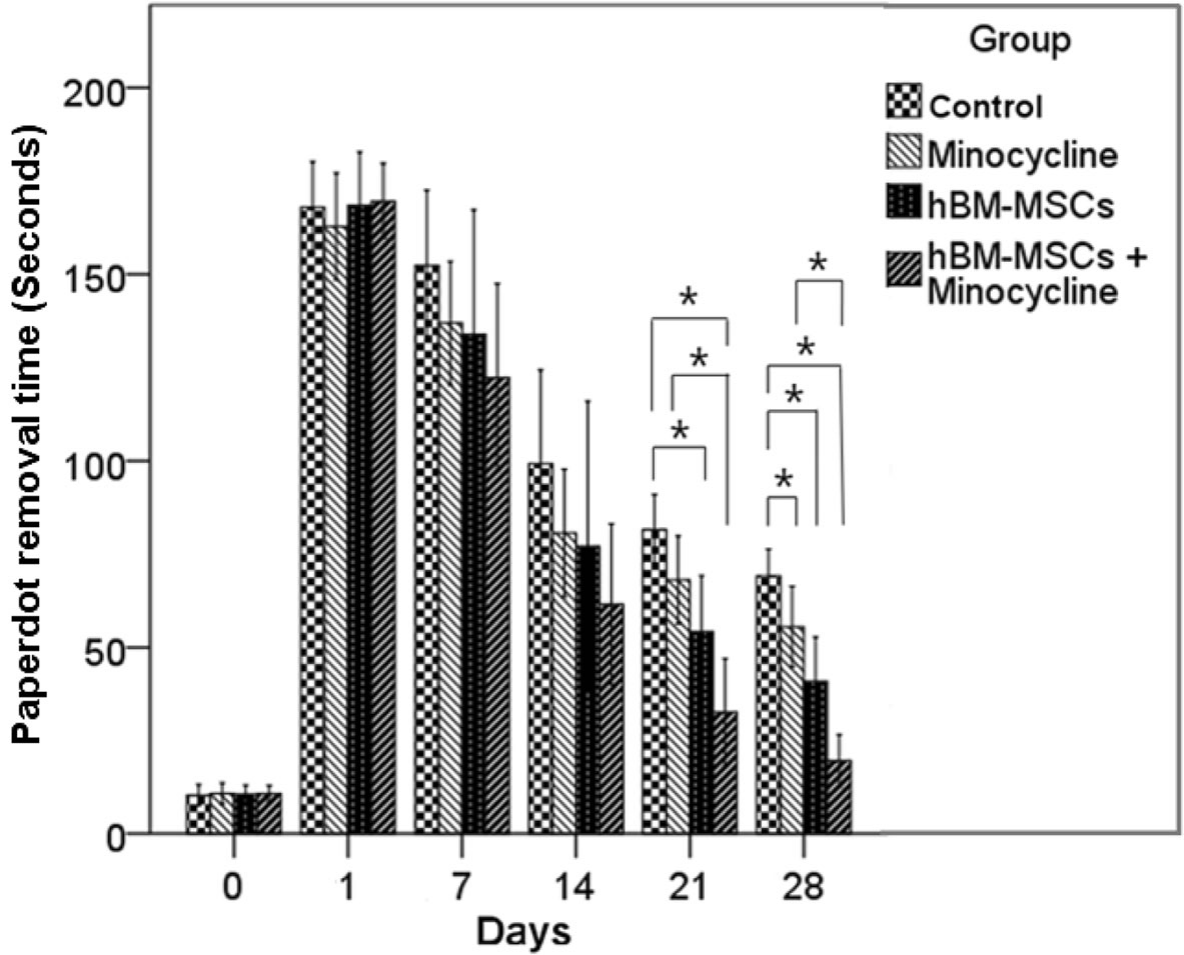
Adhesive-removal test. Results of the adhesive-removal test before middle cerebral artery occlusion (MCAO) and 1, 7, 14, 21, and 28 days after MCAO. At 21 days after MCAO, the combination group showed significant improvement compared with the control and minocycline groups. The human bone marrow–derived mesenchymal stem cell (hBM-MSC) group showed significant improvement compared with the control group. After 28 days, the minocycline group also showed significant improvement compared with the control group. *P <0.05

The adhesive-removal test also did not demonstrate significant difference between the four groups until 14 days after MCAO. At 21 days after MCAO, the combination group (32.6 ± 7.2 s) showed a significantly shorter removal time compared with the control (81.6 ± 4.7 s) and minocycline (68.0 ± 5.8 s) groups. The hBM-MSC group (68.0 ± 5.8 s) showed significantly shorter removal time compared with the control group, but there was no difference compared with the minocycline group. At day 28, the combination group (19.5 ± 3.5 s) showed significantly shorter removal time compared with the control (69.1 ± 3.6 s), minocycline (55.5 ± 5.4 s), and hBM-MSC (40.8 ± 6.0 s) groups. The hBM-MSC group also showed significantly shorter removal time compared with the control group (Fig. 2, Additional file 2: Table S2).

#### Modified Neurological Severity Score

**Fig. 3:**
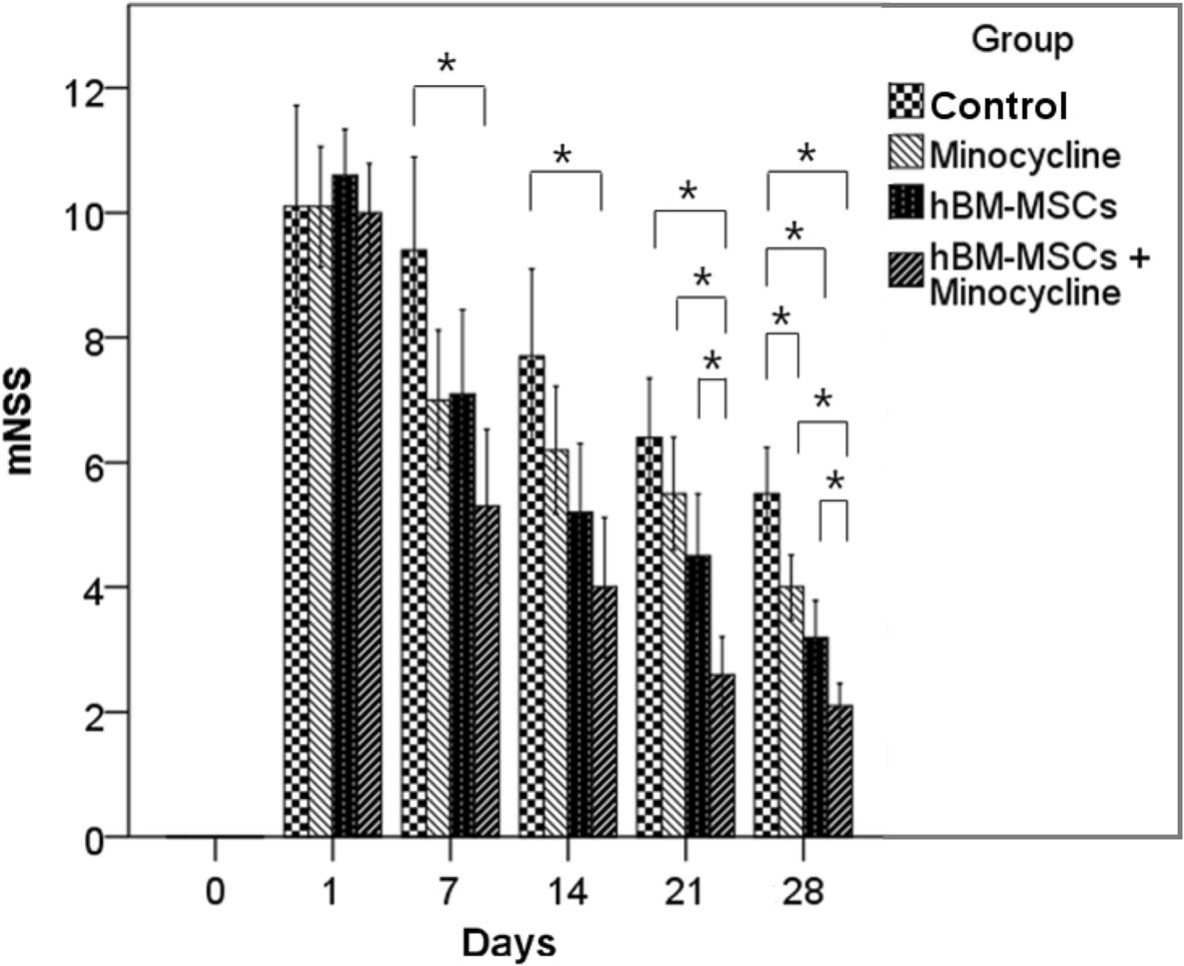
Modified neurological severity score (mNSS). mNSS before middle cerebral artery occlusion (MCAO) and 1, 7, 14, 21, and 28 days after MCAO. The combination therapy group showed significant improvement in mNSS from day 7 compared with the control group. After 21 days, the combination group showed significant improvement in mNSS compared with the minocycline and human bone marrow–derived mesenchymal stem cell (hBM-MSC) groups. At day 28, the hBM-MSC and minocycline groups also showed significant improvement compared with the control group. *P <0.05

mNSS was significantly lower in the combination group (5.3 ± 0.6) compared with the control group (9.4 ± 0.7) at day 7. This significant difference continued to day 14. By 21 days after MCAO, the combination group (2.6 ± 0.3) showed significantly lower mNSS compared with the control (6.4 ± 0.5), minocycline (5.5 ± 0.5), and hBM-MSC (2.6 ± 0.3) groups. At day 28, the mNSS of the combination group (2.1 ± 0.2) was significantly lower than those of the control (5.5 ± 0.4), minocycline (4.0 ± 0.3) and hBM-MSC (3.2 ± 0.3) groups. The mNSS values of the hBM-MSC and minocycline groups were also significantly lower than the mNSS of the control group (Fig. 3, Additional file 3: Table S3).

### Volume of infarction

**Fig. 4:**
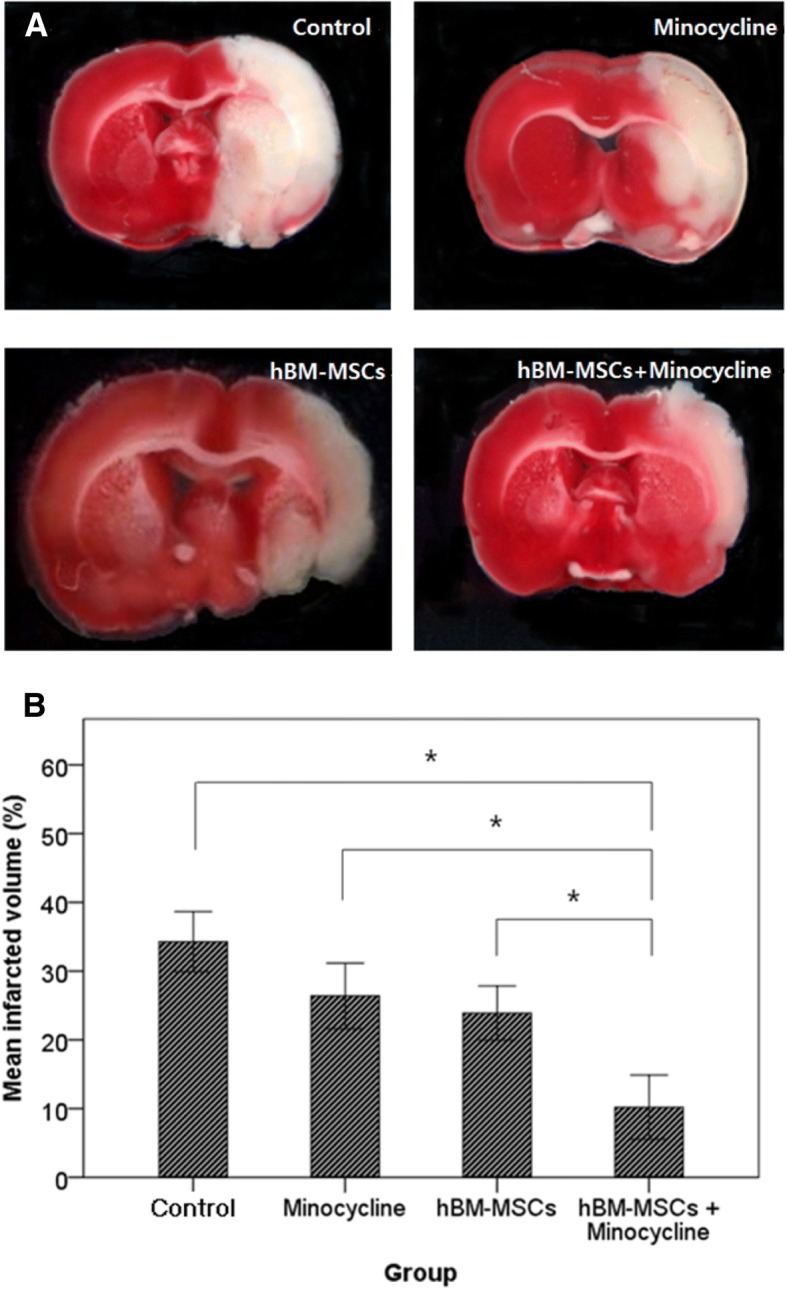
Volume of infarcted area. **a** Coronally sectioned brain tissue at a level of 10 mm from bregma with triphenyl tetrazolium chloride stain. **b** Quantitative analysis of infarction volume. The combination therapy group showed significantly lower infarction volume compared with other groups. Kruskal–Wallis test. *P <0.01. Abbreviation: *hBM-MSC* human bone marrow–derived mesenchymal stem cell

We compared the volume of infarcted areas of sacrificed rat brain sections between PBS, minocycline, hBM-MSC, and minocycline with hBM-MSC groups on day 28. The volume of infarction in the combination group (10.2 ± 2.3%) was significantly lower than in the control (34.2 ± 2.2%), minocycline (26.4 ± 2.39%), and hBM-MSC (23.9 ± 2.0%) groups (Fig. 4a, b, Additional file 4: Table S4, Additional file 5: Figure S1).

### NeuN and VEGF expression

**Fig. 5:**
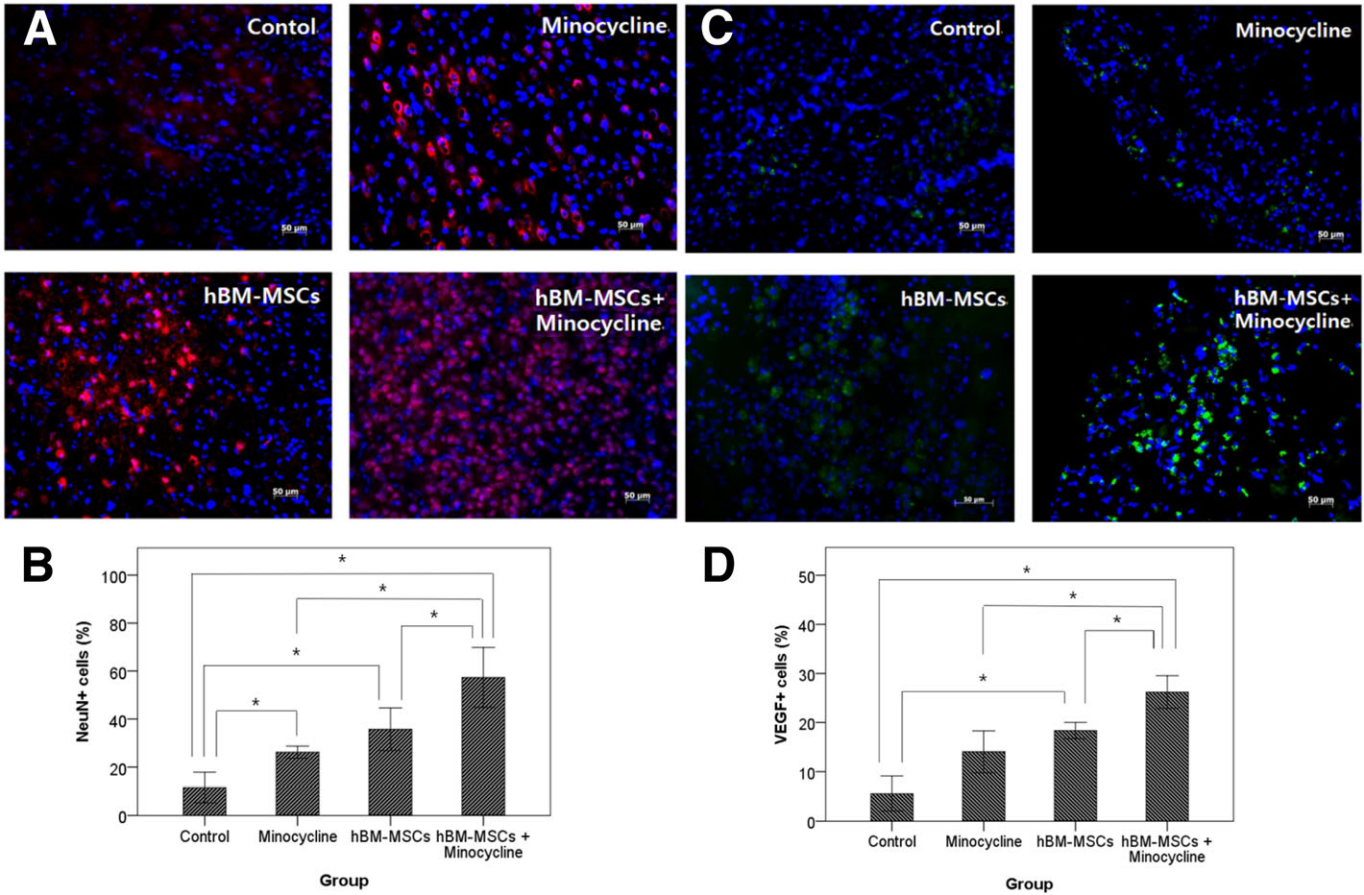
Neuronal nuclear antigen (NeuN) and vascular endothelial growth factor (VEGF) expression in the ischemic brain. **a** NeuN-positive cells in the ischemic boundary zone immunolabeled with red fluorescence. Scale bar = 50 um. **c** VEGF-positive cells in the ischemic boundary zone with immunolabeled green fluorescence. Nuclei were counterstained with 4–6-diamidino-2-phenyindole (DAPI) (blue). Scale bar = 50 um. **b** Quantitative analysis of NeuN-positive cells. **d** Quantitative analysis of VEGF-positive cells. Data are presented as mean. Kruskal–Wallis test, *P <0.01. Abbreviation: *hBM-MSC* human bone marrow–derived mesenchymal stem cell

By 28 days after MCAO, the ratio of NeuN-positive cells to total cells in the ischemic boundary zone was significantly higher in the combination group (57.3 ± 4.5%) compared with the control (11.6 ± 2.3%), minocycline (26.1 ± 0.9%), and hBM-MSC (35.7 ± 3.2%) groups. The minocycline and hBM-MSC groups also showed a significantly higher ratio of NeuN-positive cells compared with the control group (Fig. 5a, b). The ratio of VEGF-positive cells to total cells was significantly higher in the combination therapy group (26.1 ± 1.2%) compared with the control (5.6 ± 1.3%), minocycline (14.0 ± 1.5%), and hBM-MSC (18.4 ± 0.6%) groups. The hBM-MSC group had a significantly higher ratio of VEGF-positive cells compared with the control group but not with the minocycline group (Fig. 5c, d).

## Discussion

The therapeutic effects of MSCs and minocycline in treating ischemic stroke have recently indicated their use as a new fundamental treatment. Our study demonstrated that minocycline administration and transplantation of hBM-MSCs enhanced functional recovery, reduced infarction volume, and promoted expression of NeuN and VEGF. Furthermore, combination therapy demonstrated better therapeutic effects compared with monotherapy.

Only one study has demonstrated an effect of combination therapy with BM-MSCs and minocycline in rats with cerebral ischemia [29]. However, that study used a rat stroke model generated by cutting the right common carotid artery, which might induce imperfect cerebral ischemia due to collateral vessels. In addition, BM-MSCs were collected from rat bones, not humans, and neurological motor functions were evaluated with Bederson’s score ratio for only 14 days. In the present study, we obtained a stroke model with selective MCAO through an intraluminal method [26] and ensured complete occlusion of the vessel with transcranial Doppler. We also transplanted stem cells derived from human rather than rat bone marrow and evaluated neurological functions in motor, sensory, balance, reflex, and coordination through diverse neurological assessments for 28 days [30].

The optimal timing and dose of MSC transplantation are important factors in treating cerebral ischemia. The permeability of the BBB plays an important role in intravascular MSC transplantation. In ischemic brain tissue, BBB permeability due to destruction increases just after occlusion of the cerebral artery. The permeability reaches a first peak after a few hours and decreases steadily. After 48 to 72 h of arterial occlusion, the permeability of the BBB reaches a second peak, which lasts for 4 to 5 weeks [12, 31]. In the present study, we transplanted hBM-MSCs with a dose of 1.5 × 10^6^ cells in 100 μL PBS intravenously at 72 h after MCAO, which is during the second peak of BBB permeability.

An adequate dose of minocycline is also an important factor in treating cerebral ischemia. Minocycline administration in a cerebral ischemia model improves the cell viability of neurons, inhibits apoptosis of neuronal cells, ameliorates stroke-induced behavioral deficits, reduces infarction volume, and protects neurons in the penumbra. Some studies have shown that 20 mg/kg of minocycline in an MCAO model yields better functional recovery and reduces infarction volume but that 100 mg/kg aggravates neurological outcomes and infarction volume compared with controls because of the toxicity of minocycline. A minocycline overdose may compromise the BBB, allowing inflammatory cells to penetrate the CNS and deteriorating neurological deficits. Furthermore, high-dose minocycline does not elevate Bcl-2 expression but increases caspase 3/7 activity, inducing an increase of infarction volume [32]. In the present study, we administered 20 mg/kg of minocycline for 14 days as a therapeutic dose.

When MSCs are transplanted in an ischemic stroke model, they migrate to the ischemic lesion and secrete neurotropic factors, which may enhance endogenous repair mechanisms like neural regeneration, synaptogenesis, astrocyte inactivation, and immunomodulation [8]. These mechanisms repair neural networks and reconstruct neural circuits, resulting in functional recovery. The minocycline also promotes functional recovery by neuroprotection and influencing spontaneous repair-related alternative activation of microglia/macrophages [33]. In the present study, the combination therapy group showed better functional recovery compared with the monotherapy and control groups. The combination therapy group started to show significant improvement in both rotarod and adhesive-removal tests after 21 days. mNSS improvement occurred earlier, at 7 days after MCAO. The rotarod and adhesive-removal tests showed functional improvement in sensorimotor neurologic impairment, but mNSS detailed the extent of neurologic deficits in more detail, including balance and reflex [34]. Based on these results, we posit that fine neurological recovery starts at 7 days after MCAO and might manifest as concrete functional improvement after 21 days in the combination therapy group. Most functional outcomes in the single-therapy group showed significant improvement after day 28 compared with the control group, except for the adhesive-removal test in the hBM-MSC group, which showed significant improvement at day 21.

In addition to their respective therapeutic effects, the combination of minocycline and hBM-MSCs might yield synergistic effects. A few mechanisms of neurological recovery enhancement have been suggested for combination therapy. First, minocycline can contribute to a higher survival rate of transplanted stem cells. Rueger et al. reported the direct positive effect of minocycline on the survival of neural stem cells in specific concentrations *in vitro*, as well as increased activity of neural stem cells in the subventricular zone and hippocampus *in vivo*, which can enhance neurologic recovery [35]. Second, minocycline mitigates the gliogenic effect of pro-inflammatory cytokines on neural stem cells. Pro-inflammatory cytokines such as tumor necrosis factor-alpha (TNF-α) and interleukin-1 beta (IL-1β) promote differentiation of neural stem cells into astroglia, which might result in a decline of regenerative capacity. Minocycline does not affect neural stem cell proliferation directly but rather promotes proliferation through modulation of pro-inflammatory cytokines and inhibition of expression of nuclear factor-kappa B (NF-κB) [36]. The degree of functional recovery depends on the number of viable stem cells that reach the ischemic brain tissue [37]. Thus, mobilization of the stem cells may result in better neurological functional recovery [38]. These mechanisms may have enhanced neurological functional recovery in the combination therapy group.

The volume of infarction was markedly lower in the combination therapy group. Transplanted MSCs decrease infarction volume through diverse mechanisms, such as secreting neurotropic factors that enhance neuroprotection and anti-inflammatory effects [11]. Minocycline reduces expression of IL-1β and TNF-α, which are highly expressed in ischemic brain tissue, resulting in decreased volume of infarction. In the present study, each monotherapy group showed decreased infarction volume compared with the control group, but the differences were not statistically significant. The combination of minocycline and hBM-MSCs, however, yielded a significant decrease in volume of infarction compared with the monotherapy and control groups.

To date, the primary mechanisms of action by which hBM-MSCs affect neurogenesis in ischemic brain recovery remain unclear. Three main models have been proposed: transdifferentiation, cell fusion, and secretion of trophic factors [39]. A combination of these three factors may activate neurogenesis and neuroprotection of ischemia brain tissue. Minocycline administration in the acute phase of infarction also enhances neurogenesis by protecting stroke-induced newborn neurons, promoting survival of newly divided cells [40]. These mechanisms lead to high expression of NeuN-positive cells. In the present study, the combination of MSCs and minocycline led to higher expression of NeuN-positive cells than in the monotherapy groups. hBM-MSCs may promote neuronal generation by transdifferentiation, cell fusion, and secretion of neurotrophic factors, and minocycline protects stroke-induced newborn neurons generated by the effects of hBM-MSCs. In the present study, we did not track transplanted hBM-MSCs after transplantation and were not able to determine whether increased expression of NeuN was due to direct endocrine and transdifferentiation effects of hBM-MSCs or other indirect mechanisms. However, hBM-MSC transplantation in an ischemic stroke model showed a definite increase of NeuN expression in our study. The minocycline injection also increased expression of NeuN in brain cells. The mechanism behind the neurorestorative effect of minocycline may be related to the inhibition of activated microglia expressing high-mobility group box 1, lower matrix metallopeptidase 9 (MMP-9)-mediated infiltration of leukocytes, and higher levels of anti-inflammatory factors [41]. We hypothesize that, through combination therapy for ischemic stroke, transplanted hBM-MSCs might induce higher expression of NeuN and that minocycline might create favorable conditions for NeuN expression through a neuroprotective effect.

VEGF is the angiogenesis and vascular permeability factor that promotes transcriptional and post-transcriptional induction by hypoxia and ischemia [42, 43]. Angiogenesis is the key point of recovery in the ischemic penumbra because of reversibility of neuronal damage in the penumbra. The hypoxic ischemia of neurons can be recovered by restoring blood supply. When hBM-MSCs are transplanted intravenously in a cerebral ischemia rat model, they migrate to ischemic lesions, penetrate the BBB, and secrete VEGF to increase the level in ischemic brain tissue [44]. The secreted VEGF induces angiogenesis, stimulates peritubular capillary proliferation, mobilizes progenitor cells, and shows anti-apoptotic effects. Minocycline administration also enhances VEGF expression. Cai et al. showed a significant increase of VEGF expression with minocycline administration in MCAO rats [33]. In the present study, both hBM-MSC and minocycline monotherapy groups had an increase of VEGF-positive cells compared with the control group. Furthermore, the combination of hBM-MSCs and minocycline led to higher expression of VEGF-positive cells compared with single-therapy groups. The transplanted hBM-MSCs might directly secrete VEGF or induce VEGF secretion of other neuronal cells through diverse endocrine effects, and minocycline might enhance VEGF secretion in transplanted MSCs and host neuronal cells. These mechanisms could have amplified VEGF expression in the combination therapy group.

Xenogeneic transplantation of BM-MSCs can induce several adverse effects, such as tumorigenesis, graft-versus-host reactions, and infection due to immunosuppression. During the experimental period, we measured body temperature, heart rate, and weight of the rats daily. After sacrificing the rat, gross examination of visceral organs was performed. There was no evidence of tumor occurrence on pathologic evaluation through brain tissue staining and gross examination of visceral organ, but our observation period was only 4 weeks, a short period for tumorigenesis. There were some graft-versus-host reactions in the hBM-MSC transplanted group. Of 20 hBM-MSC transplanted rats, two in the hBM-MSC group and one in the combination group showed a slight increase of body temperature up to 38 °C at day 2 but this normalized after one or two days and did not progress to severe irreversible graft-versus-host reactions or infections. There were some skin rashes in two of the hBM-MSC group and two of the combination group at day of injection but these regressed the next day. There was no significant difference in heart rate and weight between rats with hBM-MSC transplantation and without hBM-MSC transplantation during the experimental period.

Treatment of ischemic stroke with hBM-MSC transplantation can be applied to humans. Numerous clinical trials have been conducted to prove the safety and efficacy of hBM-MSC transplantation in patients with ischemic stroke, with decreased infarction volume and improved neurological functions without any severe irreversible adverse effects [45–47]. Minocycline administration in patients with ischemia has also been conducted in many studies. Some found no improvement in neurological status [48], but others demonstrated a positive effect of minocycline administration without severe adverse effects [24]. For safety verification of minocycline administration in patients with ischemic stroke, a minocycline dose-finding study (MINOS trial) was conducted and showed tolerable doses up to 10 mg/kg [49]. Thus, a positive effect of hBM-MSC transplantation and minocycline administration in patients with ischemic stroke has been verified through many clinical trials. Given that a combination of these two agents yielded an amplified therapeutic effect in our ischemic stroke rat model, this combination therapy shows potential for clinical application.

There are some limitations of this study. First, only levels of NeuN and VEGF expression were evaluated, which is not sufficient to prove neurogenesis or the angiogenic effects of hBM-MSC and minocycline therapy. Second, we did not track transplanted hBM-MSCs after transplantation and were unable to determine whether successful migration of hBM-MSCs into the ischemic zone occurred. Third, we did not evaluate the laboratory findings of the blood after transplantation of hBM-MSCs to investigate adverse effects of hBM-MSC transplantation.

## Conclusions

Combination therapy of minocycline and hBM-MSCs in MCAO rats enhanced functional recovery, reduced infarction volume, and promoted higher expression of NeuN and VEGF in the ischemic boundary zone. Furthermore, combination therapy of minocycline and hBM-MSCs in MCAO rats showed amplified therapeutic effects compared with minocycline or hBM-MSCs alone.

## Additional files

Additional file 1: **Table S1.** Raw data of rotarod test, second (percentage of recovery). (DOCX 22 kb)

Additional file 2: **Table S2.** Raw data of adhesive-removal test, second. (DOCX 21 kb)

Additional file 3: **Table S3.** Raw data of modified neurological severity score (mNSS). (DOCX 21 kb)

Additional file 4: **Table S4.** Raw data of volume of infarction. (DOCX 17 kb)

Additional file 5: **Figure S1.** Raw data of triphenyl tetrazolium chloride (TCC) stain. (DOCX 821 kb)

## Abbreviations

BBB: Blood–brain barrier
CIC: Catholic Institute of Cell Therapy
CIV: Corrected infarcted volume
CNS: Central nervous system
DAPI: 4–6-diamidino-2-phenyindole
GMP: Good Manufacturing Practice
hBM-MSC: Human bone marrow–derived mesenchymal stem cell
IL-1β: Interleukin-1 beta
MCA: Middle cerebral artery
MCAO: Middle cerebral artery occlusion
mNSS: Modified neurological severity score
MSC: Mesenchymal stem cell
NeuN: Neuronal nuclear antigen
PBS: Phosphate-buffered saline
RBC: Red blood cell
TNF-α: Tumor necrosis factor-alpha
VEGF: Vascular endothelial growth factor

## References

[1] Shinozuka K, Dailey T, Tajiri N, Ishikawa H, Kaneko Y, Borlongan CV (2013). Stem cell transplantation for neuroprotection in stroke. Brain Sci.

[2] Li Y, Chen J, Chen XG, Wang L, Gautam SC, Xu YX (2002). Human marrow stromal cell therapy for stroke in rat: Neurotrophins and functional recovery. Neurology.

[3] Sammali E, Alia C, Vegliante G, Colombo V, Giordano N, Pischiutta F (2017). Intravenous infusion of human bone marrow mesenchymal stromal cells promotes functional recovery and neuroplasticity after ischemic stroke in mice. Sci Rep.

[4] Li WY, Choi YJ, Lee PH, Huh K, Kang YM, Kim HS (2008). Mesenchymal stem cells for ischemic stroke: changes in effects after ex vivo culturing. Cell Transplant.

[5] Tate CC, Fonck C, McGrogan M, Case CC (2010). Human mesenchymal stromal cells and their derivative, SB623 cells, rescue neural cells via trophic support following in vitro ischemia. Cell Transplant.

[6] Zhao Y, Lai W, Xu Y, Li L, Chen Z, Wu W (2013). Exogenous and endogenous therapeutic effects of combination Sodium Ferulate and bone marrow stromal cells (BMSCs) treatment enhance neurogenesis after rat focal cerebral ischemia. Metab Brain Dis.

[7] Mitkari B, Nitzsche F, Kerkela E, Kuptsova K, Huttunen J, Nystedt J (2014). Human bone marrow mesenchymal stem/stromal cells produce efficient localization in the brain and enhanced angiogenesis after intra-arterial delivery in rats with cerebral ischemia, but this is not translated to behavioral recovery. Behav Brain Res.

[8] Li G, Yu F, Lei T, Gao H, Li P, Sun Y (2016). Bone marrow mesenchymal stem cell therapy in ischemic stroke: mechanisms of action and treatment optimization strategies. Neural Regen Res.

[9] Wang Y, Deng Y, Zhou GQ (2008). SDF-1alpha/CXCR4-mediated migration of systemically transplanted bone marrow stromal cells towards ischemic brain lesion in a rat model. Brain Res.

[10] Song M, Mohamad O, Gu X, Wei L, Yu SP (2013). Restoration of intracortical and thalamocortical circuits after transplantation of bone marrow mesenchymal stem cells into the ischemic brain of mice. Cell Transplant.

[11] Wakabayashi K, Nagai A, Sheikh AM, Shiota Y, Narantuya D, Watanabe T (2010). Transplantation of human mesenchymal stem cells promotes functional improvement and increased expression of neurotrophic factors in a rat focal cerebral ischemia model. J Neurosci Res.

[12] Belayev L, Busto R, Zhao W, Ginsberg MD (1996). Quantitative evaluation of blood-brain barrier permeability following middle cerebral artery occlusion in rats. Brain Res.

[13] Zhang L, Li Y, Zhang C, Chopp M, Gosiewska A, Hong K (2011). Delayed administration of human umbilical tissue-derived cells improved neurological functional recovery in a rodent model of focal ischemia. Stroke.

[14] Leu S, Lin YC, Yuen CM, Yen CH, Kao YH, Sun CK (2010). Adipose-derived mesenchymal stem cells markedly attenuate brain infarct size and improve neurological function in rats. J Transl Med.

[15] Meisel R, Zibert A, Laryea M, Gobel U, Daubener W, Dilloo D (2004). Human bone marrow stromal cells inhibit allogeneic T-cell responses by indoleamine 2,3-dioxygenase-mediated tryptophan degradation. Blood.

[16] Doeppner TR, Kaltwasser B, Teli MK, Bretschneider E, Bähr M, Hermann DM (2014). Effects of acute versus post-acute systemic delivery of neural progenitor cells on neurological recovery and brain remodeling after focal cerebral ischemia in mice. Cell Death Dis.

[17] Hou Y, Ryu CH, Park KY, Kim SM, Jeong CH, Jeun SS (2013). Effective combination of human bone marrow mesenchymal stem cells and minocycline in experimental autoimmune encephalomyelitis mice. Stem Cell Res Ther.

[18] Fox C, Dingman A, Derugin N, Wendland MF, Manabat C, Ji S (2005). Minocycline confers early but transient protection in the immature brain following focal cerebral ischemia-reperfusion. J Cereb Blood Flow Metab.

[19] Lin S, Zhang Y, Dodel R, Farlow MR, Paul SM, Du Y (2001). Minocycline blocks nitric oxide-induced neurotoxicity by inhibition p38 MAP kinase in rat cerebellar granule neurons. Neurosci Lett.

[20] Yrjanheikki J, Tikka T, Keinanen R, Goldsteins G, Chan PH, Koistinaho J (1999). A tetracycline derivative, minocycline, reduces inflammation and protects against focal cerebral ischemia with a wide therapeutic window. Proc Natl Acad Sci U S A.

[21] Tikka T, Fiebich BL, Goldsteins G, Keinanen R, Koistinaho J (2001). Minocycline, a tetracycline derivative, is neuroprotective against excitotoxicity by inhibiting activation and proliferation of microglia. J Neurosci..

[22] Sanchez Mejia RO, Ona VO, Li M, Friedlander RM (2001). Minocycline reduces traumatic brain injury-mediated caspase-1 activation, tissue damage, and neurological dysfunction. Neurosurgery.

[23] Yang Y, Candelario-Jalil E, Thompson JF, Cuadrado E, Estrada EY, Rosell A (2010). Increased intranuclear matrix metalloproteinase activity in neurons interferes with oxidative DNA repair in focal cerebral ischemia. J Neurochem.

[24] Fagan SC, Cronic LE, Hess DC (2011). Minocycline development for acute ischemic stroke. Transl Stroke Res.

[25] Zheng Y, Xu L, Yin J, Zhong Z, Fan H, Li X (2013). Effect of minocycline on cerebral ischemia-reperfusion injury. Neural Regen Res.

[26] Longa EZ, Weinstein PR, Carlson S, Cummins R (1989). Reversible middle cerebral artery occlusion without craniectomy in rats. Stroke.

[27] Schallert T, Kozlowski DA, Humm JL, Cocke RR (1997). Use-dependent structural events in recovery of function. Adv Neurol.

[28] Zhao MZ, Nonoguchi N, Ikeda N, Watanabe T, Furutama D, Miyazawa D (2006). Novel therapeutic strategy for stroke in rats by bone marrow stromal cells and ex vivo HGF gene transfer with HSV-1 vector. J Cereb Blood Flow Metab.

[29] Bilen S, Pinarli F, Ak F, Fadillioglu E, Albayrak A, Boyuk G (2013). Treatment efficacy with bone marrow derived mesenchymal stem cells and minocycline in rats after cerebral ischemic injury. Stem Cell Rev.

[30] Hicks A, Schallert T, Jolkkonen J (2009). Cell-based therapies and functional outcome in experimental stroke. Cell Stem Cell.

[31] Greenwood J (1991). Mechanisms of blood-brain barrier breakdown. Neuroradiology.

[32] Matsukawa N, Yasuhara T, Hara K, Xu L, Maki M, Yu G (2009). Therapeutic targets and limits of minocycline neuroprotection in experimental ischemic stroke. BMC Neurosci.

[33] Cai Z, Yan Y, Yu C, Zhang J (2008). Minocycline inhibits neuroinflammation and enhances vascular endothelial growth factor expression in a cerebral ischemia/reperfusion rat model. Neural Regen Res.

[34] Eckert MA, Vu Q, Xie K, Yu J, Liao W, Cramer SC (2013). Evidence for high translational potential of mesenchymal stromal cell therapy to improve recovery from ischemic stroke. J Cereb Blood Flow Metab.

[35] Rueger MA, Muesken S, Walberer M, Jantzen SU, Schnakenburg K, Backes H (2012). Effects of minocycline on endogenous neural stem cells after experimental stroke. Neuroscience.

[36] Vay SU, Blaschke S, Klein R, Fink GR, Schroeter M, Rueger MA (2016). Minocycline mitigates the gliogenic effects of proinflammatory cytokines on neural stem cells. J Neurosci Res.

[37] Guzman R, De Los Angeles A, Cheshier S, Choi R, Hoang S, Liauw J (2008). Intracarotid injection of fluorescence activated cell-sorted CD49d-positive neural stem cells improves targeted cell delivery and behavior after stroke in a mouse stroke model. Stroke.

[38] Nakatomi H, Kuriu T, Okabe S, Yamamoto S, Hatano O, Kawahara N (2002). Regeneration of Hippocampal Pyramidal Neurons after Ischemic Brain Injury by Recruitment of Endogenous Neural Progenitors. Cell.

[39] Maltman DJ, Hardy SA, Przyborski SA (2011). Role of mesenchymal stem cells in neurogenesis and nervous system repair. Neurochem Int.

[40] Liu Z, Fan Y, Won SJ, Neumann M, Hu D, Zhou L (2007). Chronic treatment with minocycline preserves adult new neurons and reduces functional impairment after focal cerebral ischemia. Stroke.

[41] Venkat P, Shen Y, Chopp M, Chen J (2018). Cell-based and pharmacological neurorestorative therapies for ischemic stroke. Neuropharmacology.

[42] Valable S, Montaner J, Bellail A, Berezowski V, Brillault J, Cecchelli R (2005). VEGF-induced BBB permeability is associated with an MMP-9 activity increase in cerebral ischemia: both effects decreased by Ang-1. J Cereb Blood Flow Metab.

[43] Kawai T, Takagi N, Mochizuki N, Besshoh S, Sakanishi K, Nakahara M (2006). Inhibitor of vascular endothelial growth factor receptor tyrosine kinase attenuates cellular proliferation and differentiation to mature neurons in the hippocampal dentate gyrus after transient forebrain ischemia in the adult rat. Neuroscience.

[44] Lai T, Li M, Zheng L, Song Y, Xu X, Guo Y (2012). Over-expression of VEGF in marrow stromal cells promotes angiogenesis in rats with cerebral infarction via the synergistic effects of VEGF and Ang-2. J Huazhong Univ Sci Technolog Med Sci.

[45] Honmou O, Houkin K, Matsunaga T, Niitsu Y, Ishiai S, Onodera R (2011). Intravenous administration of auto serum-expanded autologous mesenchymal stem cells in stroke. Brain.

[46] Steinberg GK, Kondziolka D, Wechsler LR, Lunsford LD, Coburn ML, Billigen JB (2016). Clinical Outcomes of Transplanted Modified Bone Marrow-Derived Mesenchymal Stem Cells in Stroke: A Phase 1/2a Study. Stroke.

[47] Deng L, Peng Q, Wang H, Pan J, Zhou Y, Pan K, et al. Intrathecal Injection of Allogenic Bone Marrow-Derived Mesenchymal Stromal Cells in Treatment of Patients with Severe Ischemic Stroke: Study Protocol for a Randomized Controlled Observer-Blinded Trial. Transl Stroke Res. 2018. 10.1007/s12975-018-0634-y [Epub ahead of print]. 10.1007/s12975-018-0634-y 29796934

[48] Kohler E, Prentice DA, Bates TR, Hankey GJ, Claxton A, van Heerden J (2013). Intravenous minocycline in acute stroke: a randomized, controlled pilot study and meta-analysis. Stroke.

[49] Fagan SC, Waller JL, Nichols FT, Edwards DJ, Pettigrew LC, Clark WM (2010). Minocycline to improve neurologic outcome in stroke (MINOS): a dose-finding study. Stroke.

