# Supplementary material for: Combination therapy of human bone marrow–derived mesenchymal stem cells and minocycline improves neuronal function in a rat middle cerebral artery occlusion model
Source: Stem Cell Res Ther. 2018 Nov 9;9:309. doi: 10.1186/s13287-018-1011-1 (PMC6230290; doi:10.1186/s13287-018-1011-1)
Supplement: Supplementary file 1 — Table S1. Raw data of rotarod test, second (percentage of recovery). (DOCX 22 kb) [file 13287_2018_1011_MOESM1_ESM.docx]

|  | **Before MCAO** | **Day 1** | **Day 7** | **Day 14** | **Day 21** | **Day 28** |
| --- | --- | --- | --- | --- | --- | --- |
| **A1**  **A2**  **A3**  **A4**  **A5**  **A6**  **A7**  **A8**  **A9**  **A10** | 111  130.7  137.3  188  154  95.3  138.7  95  109  145.7 | 24.7(22.3)  1(0.8)  8.3(0.6)  77.3(41.1)  22.7(14.7)  24.7(25.9)  46.7(33.7)  54.7(57.6)  12.7(11.7)  92.7(63.6) | 35.3(31.8)  1(0.8)  21.3(15.5)  89.7(47.7)  75.3(48.9)  34.7(36.4)  58.3(42)  63.7(67.1)  15.7(14.4)  108.3(74.3) | 56(50.5)  16(12.2)  48.3(35.2)  115.7(61.5)  92(59.7)  49(51.4)  71.3(51.4)  70(73.7)  28.3(26.0)  129.3(88.7) | 77(69.4)  26.7(20.4)  64.3(46.8)  124.7(66.3)  106.3(69)  47(49.3)  68(49)  80.3(84.5)  40.7(37.3)  121.7(83.5) | 83.7(75,4)  30.7(23.5)  85.7(62.4)  113.3(60.3)  80.3(52.2)  55.3(58.1)  87.0(62.7)  82.0(86.3)  39.3(36.1)  110.7(76) |
| **Group A** | 130.5 ± 28.9 | 36.5 ± 30.5  (27.7 ± 21) | 50.3 ± 34.5  (37.9 ± 23.3) | 67.6 ± 3.2  (51.0 ± 22.3) | 75.7 ± 33.6  (57.6 ± 20.6) | 76.8 ± 27.4  (59.3± 18.7) |
| **B1**  **B2**  **B3**  **B4**  **B5**  **B6**  **B7**  **B8**  **B9**  **B10** | 180  114.7  233  104.7  77.7  121  98.7  171.7  135.3  131.7 | 49.7(27.6)  6.3(5.5)  35(15)  1(1)  76(97.8)  32.3(26.7)  25(25.3)  55(32)  28.7(21.2)  65.7(49.9) | 69.3(38.5)  27.3(23.8)  39.7(17)  12(11.5)  56.7(73)  75(62)  47.3(47.9)  65.7(38.3)  39.7(29.3)  76(57.7) | 90.7(50.4)  68.3(59.5)  76(32.6)  43.3(41.4)  64.7(83.3)  91.3(75.5)  68.3(69.2)  109(63.5)  65.3(48.3)  87.7(66.6) | 107.7(59.8)  69.7(60.8)  80.3(34.5)  55.3(52.8)  71(91.4)  101(83.5)  69(69.9)  119(69.3)  77(56.9)  105.3(80) | 139.3(77.4)  72.0(62.8)  94.7(40.6)  75.0(71.6)  72.0(92.7)  105.7(87.3)  73.7(74.6)  69.7(40.6)  85.3(63.1)  100.3(76.2) |
| **Group B** | 136.8 ± 46 | 37.5 ± 24.3  (30.2±27.4) | 50.9 ± 21.4  (39.9 ± 20.2) | 76.5 ±18.6  (59.0 ± 15.8 | 85.5 ± 21.1  (66.0 ± 20.5) | 88.7 ± 22.0  (68.7 ±17.4) |
| **C1**  **C2**  **C3**  **C4**  **C5**  **C6**  **C7**  **C8**  **C9**  **C10** | 120.7  74.7  135.7  155.3  135.3  185  134.7  131.7  122  158.7 | 12(9.9)  27.7(37.1)  89.3(65.8)  15.3(9.9)  53(39.2)  77(41.6)  47(34.9)  26.3(20)  39(32)  8.7(5.5) | 40(33.1)  70.3(94.1)  47.3(34.9)  22.3(14.4)  117.3(86.7)  68.7(37.1)  65(48.3)  48(36.4)  71(58.2)  43.7(27.5) | 57.7(47.8)  70.3(94.1)  29.7(21.9)  22.3(14.4)  149.3(110.3)  63.7(34.4)  120.3(89.3)  95.7(72.7  115.3(94.5)  81.3(51.2) | 50.7(42)  71(95)  110(81.1)  54.7(35.2)  88.3(65.3)  78.3(42.3)  121.3(90.1)  95.3(72.4)  104.7(85.8)  133(83.8) | 101.7(84.2)  73(97.7)  116.7(86)  126.0(81.1)  110.7(81.8)  131(70.8)  129(95.8)  89(67.6)  61.3(50.3)  124.3(78.3) |
| **Group C** | 135.4 ± 9.6 | 39.5 ± 27.3  (29±18.6) | 59.3 ± 25.8  (47 ± 25.6) | 80.6 ± 40.3  (63 ± 33.7) | 90.7 ± 27.4  (69.3 ± 22) | 106.2± 24.5  (79.4±13.9) |
| **D1**  **D2**  **D3**  **D4**  **D5**  **D6**  **D7**  **D8**  **D9**  **D10** | 132.3  130.7  67  206.3  109.3  146  133.3  117  176  93.7 | 3(2.3)  34(26.0)  3.7(5.5)  176(85.3)  68(62.2)  20(13.7)  32(24.0)  14.7(12.6)  49.3(28.0)  23(24.5) | 24.7(18.7)  95(72.7)  37.3(55.7)  123.7(60)  90(82.3)  56.3(38.6)  62.3(46.7)  48.3(41.3)  84.7(48.1)  73(77.9) | 118(89.2)  72.7(55.6)  45(67.2)  89(43.1)  95(86.9)  68.7(47.1)  101.3(76)  59.7(51)  109.7(62.3  74.7(79.7) | 125.3(94.7)  116.3(89)  58(86.6)  146(70.8)  98(89.7)  120.3(82.4  120(90)  82.7(70.7)  138(78,4)  78(83.2) | 121(91.5)  121(92.6)  60(89.6)  168(81.4)  101(92.4)  132.3(90.6)  125(93.8)  96.0(82.1)  169.7(96.4)  82.0(87.5) |
| **Group D** | 131.2 ± 35.3 | 42.3 ± 51  (29 ± 22.7) | 69.5 ± 29.8  (44.8 ± 22.4) | 83.4 ± 23.1  (65.8 ±16.6) | 90.9± 29.3  (83.5 ±8.1) | 97.4 ± 30.9  (89.8 ± 4.8) |
