# Supplementary material for: Combination therapy of human bone marrow–derived mesenchymal stem cells and minocycline improves neuronal function in a rat middle cerebral artery occlusion model
Source: Stem Cell Res Ther. 2018 Nov 9;9:309. doi: 10.1186/s13287-018-1011-1 (PMC6230290; doi:10.1186/s13287-018-1011-1)
Supplement: Supplementary file 2 — Table S2. Raw data of adhesive-removal test, second. (DOCX 21 kb) [file 13287_2018_1011_MOESM2_ESM.docx]

|  | **Before**  **MCAO** | **Day 1** | **Day 7** | **Day 14** | **Day 21** | **Day 28** |
| --- | --- | --- | --- | --- | --- | --- |
| **A1**  **A2**  **A3**  **A4**  **A5**  **A6**  **A7**  **A8**  **A9**  **A10** | 8.7  7.3  16.7  5.3  10.0  8.0  6.7  12.0  8.7  19.3 | 136.7  180.0  180.0  145.0  180.0  180.0  138.7  180.0  180.0  180.0 | 115.3  180.0  174.0  142.7  90.7  180.0  126.7  171.7  180.0  163.0 | 115.3  180.0  174.0  142.7  90.7  180.0  126.7  171.7  180.0  163.0 | 93.0  85.0  76.0  112.7  72.0  76.3  65.7  61.3  86.7  87.0 | 55.0  67.7  65.7  93.7  67.3  71.0  53.3  67.7  72.0  77.7 |
| **Group A** | 10.2 ± 1.4 | 168.0 ± 6.1 | 152.4 ± 10.1 | 99.2 ± 12.6 | 81.6 ± 4.7 | 69.1 ± 3.6 |
| **B1**  **B2**  **B3**  **B4**  **B5**  **B6**  **B7**  **B8**  **B9**  **B10** | 16.3  6.0  10.0  9.3  3.7  14.0  11.3  16.0  14.7  6.0 | 109.7  180.0  180.0  146.7  180.0  168.0  180.0  156.7  176.7  150.7 | 109.0  172.3  180.0  138.3  115.3  133.0  131.3  131.3  157.7  101.0 | 71.0  121.0  62.3  115.3  33.3  74.3  89.3  74.7  104.0  60.7 | 67.0  63.0  57.3  104.3  56.3  61.0  80.0  68.7  86.0  37.0 | 33.0  52.0  58.7  35.7  56.3  86.0  72.7  58.7  65.3  37.0 |
| **Group B** | 10.7 ± 1.4 | 162.8 ± 7.2 | 136.9 ± 8.3 | 80.6 ± 8.6 | 68.0 ± 5.8 | 55.5 ± 5.4 |
| **C1**  **C2**  **C3**  **C4**  **C5**  **C6**  **C7**  **C8**  **C9**  **C10** | 9.7  10.0  13.0  6.0  8.7  6.7  14.0  15.3  16.3  5.7 | 180.0  180.0  176.7  180.0  151.0  180.0  109.3  180.0  168.3  180.0 | 180.0  180.0  163.0  180.0  39.7  53.7  97.7  153.3  126.7  164.3 | 13.7  129.7  156.7  180.0  6.7  71.3  33.0  32.3  46.3  100.3 | 49.3  68.0  45.3  84.3  18.0  41.3  44.7  93.7  32.0  66.3 | 19.3  36.0  36.7  42.3  8.7  40.0  72.3  63.3  36.3  53.7 |
| **Group C** | 10.5 ± 1.2 | 168.5 ± 7.2 | 133.8 ± 16.7 | 77.0 ± 19.4 | 54.3 ± 7.4 | 40.8 ± 6.0 |
| **D1**  **D2**  **D3**  **D4**  **D5**  **D6**  **D7**  **D8**  **D9**  **D10** | 13.7  14.3  7.7  8.7  5.3  9.3  15.3  9.7  7.7  15.0 | 180.0  180.0  180.0  180.0  164.3  180.0  174.0  176.0  147.7  135.0 | 155.0  120.7  95.7  72.5  115.7  180.0  146.0  174.0  88.3  76.0 | 130.0  73.3  69.3  17.0  41.3  58.0  52.3  100.7  48.7  25.3 | 74.3  19.7  21.3  13.0  47.3  36.0  14.0  65.3  26.7  8.7 | 37.3  34.7  9.7  10.7  13.0  17.7  15.3  33.7  13.7  9.7 |
| **Group D** | 10.7 ± 1.1 | 169.7 ± 5.0 | 122.4 ± 12.5 | 61.6 ± 10.7 | 32.6 ± 7.2 | 19.5 ± 3.5 |
