# Supplementary material for: Combination therapy of human bone marrow–derived mesenchymal stem cells and minocycline improves neuronal function in a rat middle cerebral artery occlusion model
Source: Stem Cell Res Ther. 2018 Nov 9;9:309. doi: 10.1186/s13287-018-1011-1 (PMC6230290; doi:10.1186/s13287-018-1011-1)
Supplement: Supplementary file 3 — Table S3. Raw data of modified neurological severity score (mNSS). (DOCX 21 kb) [file 13287_2018_1011_MOESM3_ESM.docx]

|  | **Before**  **MCAO** | **Day 1** | **Day 7** | **Day 14** | **Day 21** | **Day 28** |
| --- | --- | --- | --- | --- | --- | --- |
| **A1**  **A2**  **A3**  **A4**  **A5**  **A6**  **A7**  **A8**  **A9**  **A10** | 0  0  0  0  0  0  0  0  0  0 | 11  15  10  9  5  11  9  10  12  9 | 11  14  9  8  5  10  9  9  11  8 | 10  12  7  5  5  8  7  6  9  8 | 7  5  6  4  8  6  5  5  7  7 | 6  5  6  3  7  5  5  5  6  7 |
| **Group A** | 0 | 10.1 ± 0.8 | 9.4 ± 0.7 | 7.7 ± 0.7 | 6.4 ± 0.5 | 5.5 ± 0.4 |
| **B1**  **B2**  **B3**  **B4**  **B5**  **B6**  **B7**  **B8**  **B9**  **B10** | 0  0  0  0  0  0  0  0  0  0 | 10  12  9  12  8  10  10  10  12  8 | 7  9  6  10  7  5  6  5  9  6 | 6  7  7  8  4  7  4  8  7  4 | 6  7  6  5  4  7  4  7  6  3 | 4  5  3  4  3  5  4  5  4  3 |
| **Group B** | 0 | 10.1 ± 0.5 | 7.0 ± 0.6 | 6.2 ± 0.5 | 5.5 ± 0.5 | 4.0 ± 0.3 |
| **C1**  **C2**  **C3**  **C4**  **C5**  **C6**  **C7**  **C8**  **C9**  **C10** | 0  0  0  0  0  0  0  0  0  0 | 11  11  10  12  8  10  10  11  11  12 | 6  7  5  10  3  7  8  8  7  10 | 5  7  6  8  2  5  3  5  5  6 | 6  6  5  7  2  5  3  3  4  4 | 3  3  4  5  2  3  3  2  3  4 |
| **Group C** | 0 | 10.6 ± 0.4 | 7.1 ± 0.7 | 5.2 ± 0.6 | 4.5 ± 0.5 | 3.2 ± 0.3 |
| **D1**  **D2**  **D3**  **D4**  **D5**  **D6**  **D7**  **D8**  **D9**  **D10** | 0  0  0  0  0  0  0  0  0  0 | 11  11  10  10  8  10  10  12  8  10 | 9  5  7  2  4  5  5  7  5  4 | 4  3  5  1  4  6  4  7  4  2 | 3  3  2  1  3  2  2  4  4  2 | 2  3  2  1  2  2  2  3  2  2 |
| **Group D** | 0 | 10.0 ± 0.4 | 5.3 ± 0.6 | 4.0 ± 0.6 | 2.6 ± 0.3 | 2.1 ± 0.2 |
