# Supplementary material for: Combination therapy of human bone marrow–derived mesenchymal stem cells and minocycline improves neuronal function in a rat middle cerebral artery occlusion model
Source: Stem Cell Res Ther. 2018 Nov 9;9:309. doi: 10.1186/s13287-018-1011-1 (PMC6230290; doi:10.1186/s13287-018-1011-1)
Supplement: Supplementary file 4 — Table S4. Raw data of volume of infarction. (DOCX 17 kb) [file 13287_2018_1011_MOESM4_ESM.docx]

|  | **Infarction volume (%)** | |  |
| --- | --- | --- | --- |
| **A1**  **A2**  **A3**  **A4**  **A5** | 41.09  32.29  34.57  28.78  33.7 | |  |
| **Group A** | 34.2 ± 2.2 | |  |
| **B1**  **B2**  **B3**  **B4**  **B5** | 37.58  26.53  25.19  25.61  21.67 | |  |
| **Group B** | 26.4 ± 2.39 | |  |
| **C1**  **C2**  **C3**  **C4**  **C5** | | 21.71  19.84  26.17  21.36  28.21 | |
| **Group C** | | 23.9 ± 2.0 | |
| **D1**  **D2**  **D3**  **D4**  **D5** | | 18.35  11.97  7.8  4.89  7.98 | |
| **Group D** | | 10.2 ± 2.3 | |
