# Supplementary figures and images for: Combination therapy of human bone marrow–derived mesenchymal stem cells and minocycline improves neuronal function in a rat middle cerebral artery occlusion model
Source: Stem Cell Res Ther. 2018 Nov 9;9:309. doi: 10.1186/s13287-018-1011-1 (PMC6230290; doi:10.1186/s13287-018-1011-1)

**
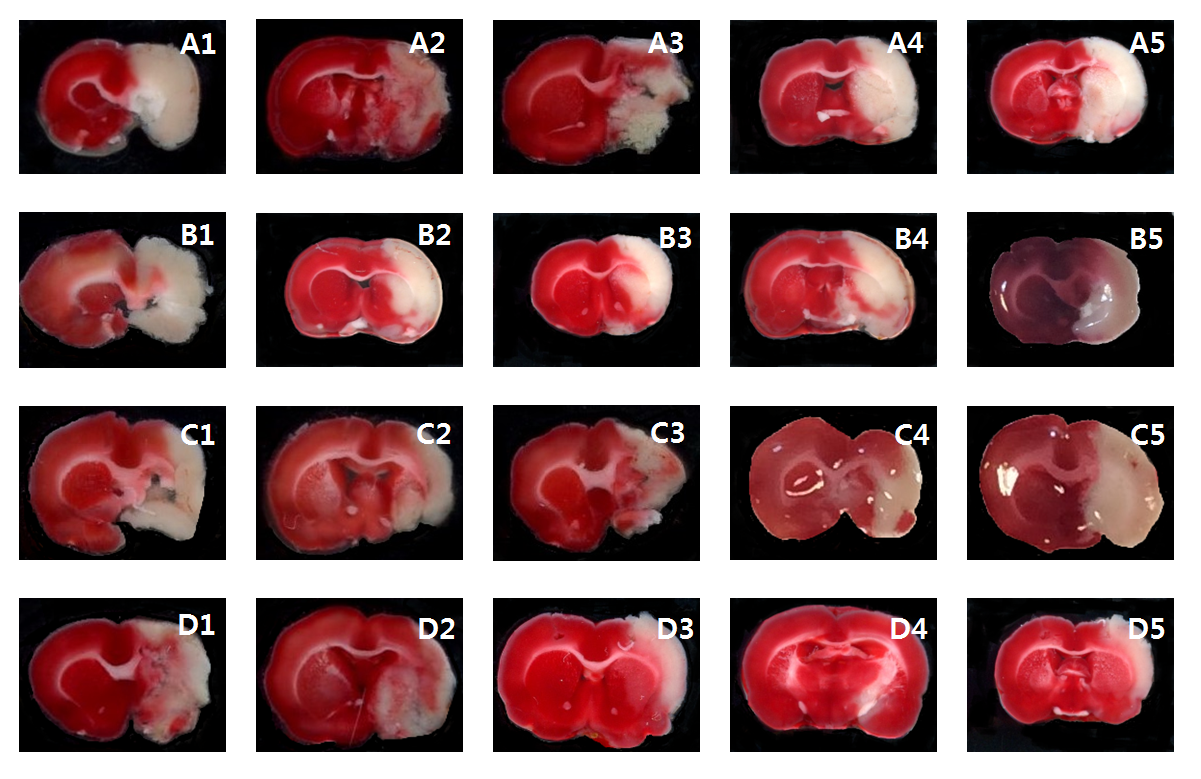
**

Supplement: Supplementary file 5 — Figure S1. Raw data of triphenyl tetrazolium chloride (TCC) stain. (DOCX 821 kb) [file 13287_2018_1011_MOESM5_ESM.docx]
